# Evaluating genomic signatures of aging in brain tissue as it relates to Alzheimer’s disease

**DOI:** 10.1038/s41598-023-41400-1

**Published:** 2023-09-07

**Authors:** Megan T. Lynch, Margaret A. Taub, Jose M. Farfel, Jingyun Yang, Peter Abadir, Philip L. De Jager, Francine Grodstein, David A. Bennett, Rasika A. Mathias

**Affiliations:** 1 Department of Medicine, School of Medicine, Johns Hopkins University, Baltimore, MD USA; 2 Department of Biostatistics, Johns Hopkins Bloomberg School of Public Health, Baltimore, MD USA; 3 Rush Alzheimer’s Disease Center, Rush University Medical Center, Chicago, IL USA; 4 Center for Translational and Computational Neuroimmunology, Department of Neurology, Columbia University Irving Medical Center, New York, NY USA

**Keywords:** Medical genomics, Computational biology and bioinformatics, Neuroscience

## Abstract

Telomere length (TL) attrition, epigenetic age acceleration, and mitochondrial DNA copy number (mtDNAcn) decline are established hallmarks of aging. Each has been individually associated with Alzheimer’s dementia, cognitive function, and pathologic Alzheimer’s disease (AD). Epigenetic age and mtDNAcn have been studied in brain tissue directly but prior work on TL in brain is limited to small sample sizes and most studies have examined leukocyte TL. Importantly, TL, epigenetic age clocks, and mtDNAcn have not been studied jointly in brain tissue from an AD cohort. We examined dorsolateral prefrontal cortex (DLPFC) tissue from N = 367 participants of the Religious Orders Study (ROS) or the Rush Memory and Aging Project (MAP). TL and mtDNAcn were estimated from whole genome sequencing (WGS) data and cortical clock age was computed on 347 CpG sites. We examined dementia, MCI, and level of and change in cognition, pathologic AD, and three quantitative AD traits, as well as measures of other neurodegenerative diseases and cerebrovascular diseases (CVD). We previously showed that mtDNAcn from DLPFC brain tissue was associated with clinical and pathologic features of AD. Here, we show that those associations are independent of TL. We found TL to be associated with β-amyloid levels (beta = − 0.15, *p* = 0.023), hippocampal sclerosis (OR = 0.56, *p* = 0.0015) and cerebral atherosclerosis (OR = 1.44, *p* = 0.0007). We found strong associations between mtDNAcn and clinical measures of AD. The strongest associations with pathologic measures of AD were with cortical clock and there were associations of mtDNAcn with global AD pathology and tau tangles. Of the other pathologic traits, mtDNAcn was associated with hippocampal sclerosis, macroscopic infarctions and CAA and cortical clock was associated with Lewy bodies. Multi-modal age acceleration, accelerated aging on both mtDNAcn and cortical clock, had greater effect size than a single measure alone. These findings highlight for the first time that age acceleration determined on multiple genomic measures, mtDNAcn and cortical clock may have a larger effect on AD/AD related disorders (ADRD) pathogenesis than single measures.

## Introduction

Aging, a progressive deterioration of physiologic reserve with time^1^, is a risk factor of many complex diseases^2^. Biologic age, determined by genomic markers of aging, may be better than chronologic age at predicting functional capacity and rate of aging^3^. Cellular and molecular hallmarks of aging include mitochondrial dysfunction, genomic instability, TL shortening, and epigenetic alterations^4^. Physiological functions, including some cognitive functions, decline with chronologic age and biological markers of aging have been linked to cognitive decline and Alzheimer’s disease (AD).

Mitochondrial dysfunction is well studied in AD and aging^5–7^. Genomic instability leads to mitochondrial DNA variations and increased age correlates with increases in mtDNA heteroplasmy and decreases in mtDNA copy number (mtDNAcn)^8,9^. Some studies have found lower mtDNAcn in brain tissue in AD that may even be region-specific^10–13^. Using quantitative PCR methods and hippocampal tissue, one study found that AD pyramidal neurons, but not dentate granule neurons, had significantly lower mtDNAcn^10^. A later study showed that mtDNAcn measurements from the frontal cortex were 28% lower in AD patients but mtDNAcn from blood samples, hippocampus, and cerebellum tissues was not different between controls and AD^11^. Similarly, in cerebellum tissue, lower mtDNAcn has been noted in AD^12^.

Epigenetic alterations, including DNA methylation (DNAm), have been linked to AD pathology and cognitive aging^14,15^. In epigenome-wide association studies of AD brain samples from several regions, differential DNAm at several loci has been related to AD pathology^16–19^. Several studies highlight the differential methylation patterns of *ANK1* in AD, including our own^15–17^. A recent study identified differential methylation of 121 genes that were associated with neuropathologies in a cross-cortex meta-analysis^20^. In addition there are several “epigenetic clocks” which are biomarkers of aging generated from DNAm states across select sites. We have previously shown that the Horvath clock and Phenoage were associated with cognitive decline and AD-related neuropathologic traits in DLPFC brain samples^21,22^. Telomere attrition has been associated with aging and numerous diseases including AD^23–26^. Studies linking telomere length (TL) to AD have largely been conducted in leukocyte cells^27–31^, and the role of TL in brain tissue in AD has only previously been examined in cohorts with small sample sizes^32,33^.

In the Religious Orders Study and the Rush Memory and Aging Project (ROSMAP) data, two signatures of biological aging in the brain were previously studied. We found that dorsolateral prefrontal cortex (DLPFC) mtDNAcn is nearly 10% lower in pathologic AD relative to non-AD participants^34^. We previously reported associations between mtDNAcn and clinical measures of AD including lower global cognitive function and greater cognitive decline. We also reported a modest association between mtDNAcn and higher quantitative global AD pathology score and higher tau^34^. Recently, a “Cortical” clock trained in human cortex tissue was developed which was more strongly associated with AD diagnosis and β-amyloid than other clocks^35^. Cortical clocks were assessed in DLPFC brain samples from ROSMAP data and each standard deviation higher cortical clock age was related to 90% greater likelihood of pathologic AD. Higher cortical clock age was associated with clinical dementia-related phenotypes and quantitative AD traits including global AD pathology, tau tangle density, and β-amyloid^36^.

Here, we introduce a third genomic predictor of biologic age in the same set of samples, TL, and expand on prior findings from the individual measures of biological age by determining multimodal age acceleration—i.e., accelerated aging across epigenetic cortical clock age, brain mtDNAcn and brain TL. We bioinformatically estimated TL from whole exome sequencing data, generated from ROSMAP DLPFC brain samples, and leveraged the existing mtDNAcn and epigenetic cortical clock estimates of the same samples^34,36^ to understand the effect of multiple genomic signatures of aging on clinical and pathologic features of AD, other neurodegenerative diseases, and cerebrovascular disease (CVD).

## Methods

### Cohorts

ROS includes older priests, nuns, and brothers from across the US^37,38^. MAP includes older men and women from across the Chicago metropolitan area^39^. These two cohort studies of aging and dementia share common clinical and post-mortem data collection at the item level allowing data to be merged. Participants entered these studies without known dementia and agreed to annual clinical and cognitive assessments and brain donation after death. Both studies were approved by an Institutional Review Board of Rush University Medical Center and all experiments were performed in accordance with university guidelines. All participants signed an informed consent, an Anatomical Gift Act for organ donation, and a repository consent allowing their data to be shared.

### Clinical evaluation

Diagnosis of dementia and mild cognitive impairment (MCI) were based on a summary diagnostic opinion by a neurologist with expertise in dementia after death and was made blinded to postmortem data^40^. Raw scores for overall cognitive function were generated from 19 cognitive tests. These were converted to Z scores and averaged to yield global cognition. 38 Cognitive decline is the person-specific rate of change in global cognition over time. It is estimated from a linear mixed effects effect model that controls for age at baseline, sex, and years of eduction^41^.

### Neuropathologic evaluation

The NIA-Reagan diagnosis of pathologic AD is based on consensus recommendations for postmortem diagnosis and the criteria rely on the distribution and density of neurofibrillary tangles and neuritic plaques^42,43^. Global AD pathology is a quantitative summary of neuritic plaques, diffuse plaques, and neurofibrillary tangles determined by microscopic examination of silver-stained slides from five brain regions (mid-hippocampus, entorhinal cortex, midfrontal cortex, middle temporal cortex, and inferior parietal cortex)^44^. Amyloid beta protein and tau tangles were assessed in 8 brain regions by molecularly specific immunohistochemistry by image analysis and stereology respectively^45^.

Other neurodegenerative disease pathologies were assessed. Presence of TDP-43 inclusions in neurons and glia was determined by immunohistochemistry of 8 brain regions and four stages of TDP-43 distribution were assigned^46^. Lewy body disease was described in four stages determined by α-synuclein distribution based on algorithm accepted criteria^47^. The presence of hippocampal sclerosis was evaluated unilaterally in a coronal section of the mid hippocampus at the level of the lateral geniculate body and graded based on severe neuronal loss and gliosis in CA1 and/or subiculum^48^.

Several indices of CVD were characterized. The presence, size and age of gross chronic infarcts were identified at the time of autopsy and verified microscopically; microscopic infarctions were identified on hematoxylin and eosin stained slides^49,50^. A semiquantitative summary of cerebral amyloid angiopathy (CAA) was assessed in four neocortical regions by β-amyloid immunostaining. Meningeal and parenchymal vessels were assessed for amyloid-β deposition in each region and scored from 0 to 4 and then averaged across the four regions^51,52^. Cerebral atherosclerosis rating was determined by visual inspection after paraformaldehyde fixation at the Circle of Willis at the base of the brain and severity was graded by visual examination.^53^ Arteriolosclerosis described as fibrohyalinotic thickening of arterioles with consequent narrowing of the vascular lumen was examined in the basal ganglia^49,50,54^.

### Whole genome sequencing

Whole-genome sequencing (WGS) was performed on DNA extracted from the DLPFC using Qiagen’s QIAamp DNA kit (n = 367) as previously described^55^. Briefly, WGS libraries were prepared using the KAPA Hyper Library Preparation Kit. DNA was sheared using a Covaris LE220 sonicator (adaptive focused acoustics). DNA fragments underwent bead-based size selection and were subsequently end repaired, adenylated, and ligated to Illumina sequencing adapters. Ligated DNA libraries were evaluated by fluorescent-based assays including qPCR with the Universal KAPA Library Quantification Kit and Fragment Analyzer (Advanced Analytics) or BioAnalyzer (Agilent 2100). Libraries were sequenced on an Illumina HiSeq X sequencer (v2.5 chemistry) using 2 × 150 bp cycles.

### Biologic age estimates from WGS data

#### Telomere length

TL was estimated bioinformatically from a single time point using WGS by Telseq. TelSeq estimates telomere length of each individual by counting the number of contiguous repeats of the telomere-identifying hexamer TTAGGG^56^. Given that most of our data was sequenced using read lengths of 151, we chose to use a repeat number of 12. Read counts are then normalized according to the number of reads in the individual WGS dataset with 48%-52% GC content. TelSeq generates an estimate of TL in bp similar to laboratory assays Southern blot21 and flowFISH and our group has previously demonstrated in detail that TelSeq estimates are highly correlated with both Southern blot and flowFISH measurements^57^.

#### mtDNAcn

Raw mtDNAcn estimates were calculated as (*cov*_mt_/*cov*_nuc_) × 2 using mtDNA and nuclear DNA from the WGS data where *cov*_nuc_ is the median sequence coverages of the autosomal chromosomes and *cov*_mt_ of the mitochondrial genome. These were calculated using R/Bioconductor (packages GenomicAlignments and GenomicRanges). Ambiguous regions were excluded using the BSgenome package. For analyses, mtDNAcn was standardized and logarithmized by methods described previously^34^.

#### Cortical clock age

DNA methylation profiles, measured in DLPFC tissue from brain samples, were generated using the Illumina Infinium Human Methylation450 platform. Preprocessing and quality control was previously described in detail^36^. The cortical clock was designed in postmortem cortical specimens to predict chronologic age from 347 CpG sites^35^.

### Estimation of neuronal proportion from RNA-seq data

Proportion of neurons were estimated as previously described^34^. Briefly, the Digital Sorting Algorithm (DSA) was applied to a set of published marker genes that were used previously to deconvolute cortical RNA-seq data^58,59^. RNA-seq samples were extracted using Qiagen’s miRNeasy mini kit (cat. No. 217004) and the RNase free DNase Set (cat. No. 79254). Sequencing was conducted on the Illumina HiSeq and NovaSeq6000. RNA-seq data were trimmed mean of M values (TMM) normalized and technical variables were regressed out. Only marker genes with a mean transcription level^3^ 2 counts per million reads mapped (cpm) were used and the median transcription level of all marker genes per cell type was calculated.

### Binary definition of age acceleration

We defined multi-modal aging by creating a binary definition of accelerated biological aging (i.e. accelerated vs. not accelerated) based on cortical clock age and mtDNAcn. For cortical clock, residuals were obtained from the regression of clock age on chronologic age. Samples with positive residual values were categorized as accelerated cortical clock age (CC_age+_) and negative residual values were categorized as non-accelerated cortical clock age (CC_age-_). Age acceleration on mtDNAcn (mtDNA_age+_) was defined as below median of the estimates; values larger than the median 50^th^ were considered not accelerated (mtDNA_age-_). Individually, biological aging measures were examined in multivariate regression models adjusting for covariates: CC_age+_ *vs.* CC_age-_ and mtDNA_age+_ vs. mtDNA_age-_. Furthermore, to examine the impact of age acceleration on multiple measures (i.e., mtDNAcn and cortical clock), we also categorized individuals using a combination of these binary biological age acceleration predictors; CC_age+/_ mtDNA_age+_ represents an individual who is age accelerated on both cortical clock and mtDNAcn and CC_age-/_ mtDNA_age-_ represents an individual who is not age accelerated on either mtDNAcn or cortical clock.

### Statistical analysis

Standard multivariable regression analysis pipelines were run in R. In the first analysis, the three biologic predictors of age (TL, mtDNAcn, and cortical clock) were the primary predictors and they were examined as quantitative measures individually in univariate and jointly in multivariate analysis models. Quantitative outcomes, (global cognition, cognitive decline, global AD pathology, amyloid, and tau) were standardized to obtain comparable effect sizes and assessed by linear regression. Binary (dementia diagnosis, cognitive impairment diagnosis, NIA-Reagan diagnosis, hippocampal sclerosis, gross chronic infarcts, and microinfarcts) and ordinal outcomes (TDP-43, Lewy bodies, cerebral amyloid angiopathy, cerebral atherosclerosis, and arteriolsclerosis) were assessed by logistic and ordinal regression models. Age at death and sex were covariates for all analyses and education was also included as a covariate when assessing clinical outcomes, i.e., dementia, MCI, global cognition, and cognitive decline. Post-mortem interval (pmi) was not included as a covariate because there was no relationship between pmi and any of the three biologic predictors of age or age at death. Therefore, we did not include pmi as a covariate in our analysis. For the multi-modal analysis CC_age+_ vs. CC_age-_, mtDNA_age+_ vs. mtDNA_age-_, and CC_age+_/ mtDNA_age+_ vs. CC_age-_/ mtDNA_age-_, the same covariates referenced above were used except age at death because the dichotomization of age acceleration was based on residuals of epigenetic age vs. age at death. For each statistical test, a significance threshold of *p*-value < 0.05 was applied.

To test departure from additivity for multi-modal models, a likelihood ratio test was performed comparing a 3-factor categorical age acceleration variable vs. an additive model with a numeric age acceleration variable. In the 3-factor model, baseline was CC_age-_/mtDNA_age-_ and two categories were each compared to the baseline group: 1 = CC_age−_/mtDNA_age+_ or = CC_age+_/mtDNA_age-_ and 2 = CC_age+_/mtDNA_age+_. In the additive model, these groups were coded as a linear variable.

### Ethical approval

All methods were carried out in accordance with relevant guidelines and regulations. All experiments were performed in accordance with university guidelines for human research and the study was approved by the Institutional Review Board of Rush University Medical Center. All participants signed an informed consent, an Anatomical Gift Act for organ donation, and a repository consent allowing their data to be shared.

## Results

### Sample characteristics

Our sample included N = 367 non-Latino white subjects from ROSMAP^28,29^. There were more females than males (64% vs 35%) and the average age at death was 88 years old (Table 1). In this sample, nearly half had dementia and about a quarter had MCI proximate to death. The *APOE* e4 allele (e4/e4 or e3/e4) was carried by 25.9% of the population. All of N = 367 study subjects had WGS generated on brain DNA, N = 258 had brain methylation data, N = 256 had brain RNASeq with N = 171 having an overlap for all three assays available.

**Table 1:** Characteristics of ROSMAP study subjects with multi-omics data available from brain DLPFC samples**.**

	All samples
N	367
Sex (male)	128 (34.9%)
Age at death (years)	88.4 ± 6.7
Whole Genomce Seq	367
Methylation profiles	258
RNA seq	256
ApoE e4 carriers (n(%))	95 (25.9%)
Dementia diagnosis	
No Dementia	195 (53.1%)
Dementia	172 (46.9%)
Cognitive Impairment diagnosis	
NCI	102 (27.8%)
CI	265 (72.2%)
Global cognition	− 1.02 ± 1.18
Cognitive decline	− 0.03 ± 0.10
NIA-Reagan diagnosis	
No AD	108 (30.5%)
AD	246 (69.5%)
Global AD pathology	0.84 ± 0.66
Amyloid	1.85 ± 1.16
Tau	2.34 ± 1.35
TDP-43	
None	172 (51.3%)
Amygdala	54 (16.1%)
Limbic	79 (23.6%)
Neocortical	30 (9.0%)
Lewy Bodies	
None	270 (76.9%)
Nigral	6 (1.7%)
Limbic	24 (6.8%)
Neocortical	51 (14.5%)
Hippocampal sclerosis	
Not present	321 (88.7%)
Present	41 (11.3%)
Gross chronic infarcts	
None	226 (61.6%)
One or more	141 (38.4%)
Microinfarcts	
None	263 (71.7%)
One or more	104 (28.3%)
Cerebral amyloid angiopathy	
None	69 (19.3%)
Mild	158 (44.1%)
Moderate	87 (24.3%)
Severe	44 (12.3%)
Cerebral atherosclerosis	
None	50 (13.7%)
Mild	165 (45.2%)
Moderate	117 (32.1%)
Severe	33 (9.0%)
Arterioloscerosis	
None	102 (27.9%)
Mild	120 (32.8%)
Moderate	103 (28.1%)
Severe	41 (11.2%)

N(%) presented for categorical variables and mean ± se for quantitative traits.

There is a difference in the set of samples used in each analysis. The analysis of mtDNAcn and TL used N = 256 samples with neuronal cell composition estimates from RNASeq data. The analysis of mtDNAcn, TL, and Cortical clock used N = 258 samples with available methylation data and cell composition was estimated from methylation. There are N = 171 samples that are a direct overlap between the two sets.

### Measures of biological aging in brain

**Figure 1:**
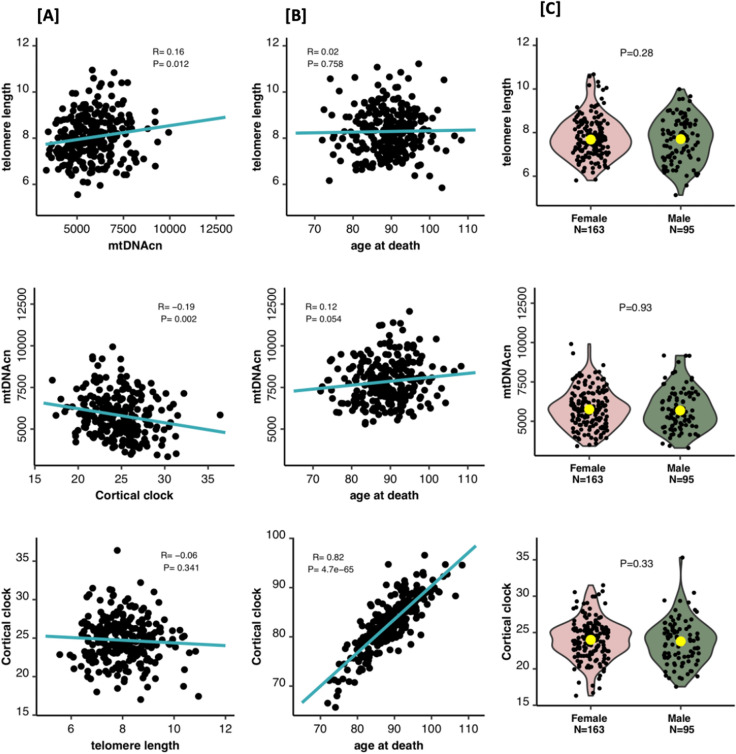
Distribution of mtDNAcn, cortical clock, and TL by gender and age. TL unit is kilobase (kb). Cortical clock unit is age in years. (**A**) Correlation between mtDNAcn, Cortical clock, and TL where each measure was adjusted for age, sex, and neuronal fraction. (**B**) Correlation between age and mtDNAcn, Cortical clock and TL, where each measure was adjusted for sex and neuronal fraction. (**C**) mtDNAcn, Cortical clock, and TL by sex, where each measure was adjusted for age and neuronal fraction. The yellow dots indicate median mtDNAcn, Cortical clock, and TL values. Note: Cortical clock is adjusted for age in panels A and C, and therefore on a different scale from panel B.

Figure 1 shows the correlation between the three measures of biological aging in the ROSMAP brain samples, and their association with age and sex. Adjusting for age, sex, and neuronal fraction, we found brain mtDNAcn and TL measures to be positively correlated (R = 0.16, *p* = 0.0123), mtDNAcn and cortical clock age to be negatively correlated (R = − 0.19, *p* = 0.0018), and no correlation between TL and cortical clock age (R = − 0.059, *p* = 0.3412). Associations between sex and TL, mtDNAcn, or cortical clock age were not significant. No significant correlation with age was noted for TL and mtDNAcn, but there was a very strong positive correlation with Cortical age (R = 0.85, *p* = 2.8e−72).

### Association between TL and clinical and pathological indices

Given that this is the first evaluation of TL in the ROSMAP brain samples, Table 2 shows the individual association between TL on a quantitative scale and clinical and neuropathologic phenotypes. Adjusting for age, sex, and neuronal fraction using cell composition derived from RNASeq on these samples, we did not find associations with any clinical traits. For AD traits, we identified an inverse association between β-amyloid levels and TL (beta = − 0.15, *p* = 0.0232). We also identified an inverse association between TL and hippocampal sclerosis (OR = 0.56, *p* = 0.0015) but a direct association with cerebral atherosclerosis (OR = 1.44, *p* = 0.0007). None of the other pathologic phenotypes were significantly associated with TL.

**Table 2:** Association of brain TL with clinical and neuropathologic phenotypes adjusting for cell composition from RNAseq data in N = 256 DLPFC brain samples and joint analysis evaluating the association of mtDNAcn, Cortical age and TL with outcomes adjusting for neuronal proportions from methylation data in N = 258 DLPFC brain samples.

Phenotype	*Modeling brain TL alone*		*Joint analysis of mtDNAcn, cortical clock, and brain TL*					
	Effect _TL_	P _TL_	Effect _mtDNAcn_	P _mtDNAcn_	Effect _clock_	P _clock_	Effect _TL_	P _TL_
Dementia diagnosis	0.94	0.6077	**0.61**	**0.0008**	1.06	0.2468	1.06	0.6780
Cognitive Impairment diagnosis	0.89	0.3948	**0.69**	**0.0318**	1.04	0.5315	0.96	0.7997
Global cognition*	0.00	0.9677	**0.32**	**1.7E−05**	− **0.06**	**0.0134**	− 0.06	0.3946
Cognitive decline*	0.00	0.8450	**0.03**	**1.8E−05**	0.00	0.0676	0.00	0.5286
NIA-Reagan diagnosis	0.97	0.7944	0.79	0.1584	**1.19**	**0.0029**	1.12	0.4652
Global AD pathology*	0.00	0.9922	− **0.07**	**0.0034**	**0.03**	**0.0003**	0.03	0.2242
Amyloid*	− **0.15**	**0.0232**	− 0.05	0.4450	**0.06**	**0.0047**	0.11	0.0910
Tau*	0.04	0.5576	− **0.30**	**0.0007**	**0.07**	**0.0183**	0.10	0.2711
TDP-43	0.89	0.2771	0.92	0.5433	1.03	0.5170	1.07	0.6056
Lewy bodies	0.88	0.3745	0.88	0.4216	**1.16**	**0.0063**	1.02	0.9001
Hippocampal sclerosis	**0.56**	**0.0015**	**1.67**	**0.0298**	1.08	0.3035	1.03	0.9133
Gross chronic infarcts	0.88	0.2655	**0.75**	**0.0374**	1.04	0.4195	0.98	0.8591
Microinfarcts	0.83	0.1443	0.84	0.2445	1.06	0.2829	1.03	0.8388
Cerebral amyloid angiopathy	1.08	0.4459	**0.78**	**0.0449**	1.02	0.5810	1.03	0.8204
Cerebral atherosclerosis	**1.44**	**0.0007**	0.81	0.0900	0.94	0.1565	1.10	0.4244
Arteriolsclerosis	0.95	0.6200	0.81	0.0867	0.94	0.1327	0.88	0.2632

Effect sizes are presented as Odds Ratios for categorical traits. For quantitative traits (indicated by *) the effect size is the β coefficient.

Each outcome was analyzed separately in a regression model adjusted for age, sex, and neuronal fraction from either RNA seq data (Modeling brain TL alone) or methylation data (Joint analysis of mtDNAcn, cortical clock, and brain TL). Clinical dementia diagnosis, MCI diagnosis, global cognition, and cognitive decline were adjusted for age, sex, neuronal fraction, and years of education. The beta values represent the change in pathologic outcomes for every 1 unit increase in TL, mtDNAcn, or Cortical clock age. The odds represent the increase or decrease in odds of moving into a higher group relative to baseline for every 1 unit increase in TL, mtDNAcn, or Cortical clock age. Associations between outcomes and mtDNAcn and Cortical clock age have been published previously in ROSMAP data^34,36^.

Significant values are in bold.

### Evaluating independence in association between three biological measures of brain aging with clinical outcomes and pathological indices

In the joint multivariate analysis in which all three estimates of biologic age were included as linear predictors in a single model for each clinical and neuropathologic phenotype (Table 2**, **Supplemental Fig. 1), we observed that mtDNAcn was associated with dementia (OR = 0.61, *p* = 0.0008), MCI (OR = 0.69, *p* = 0.0318), and both level of (beta = 0.32, *p* = 1.7 × 10^–5^) and change in cognition (beta = 0.03, *p* = 1.8 × 10^–5^). The cortical clock was inversely associated with global cognition (beta = − 0.06, *p* = 0.0134). By contrast, TL no longer remained significant for any clinical trait. Interestingly, cortical clock age was positively associated with all four pathologic measures of AD; NIA-Reagan diagnosis (OR = 1.19, *p* = 0.0029), global AD pathology (beta = 0.03, *p* = 0.0003), amyloid beta (beta = 0.06, *p* = 0.0047), and tau tangles (beta = 0.07, *p* = 0.0183). Additionally, mtDNAcn was associated with global AD pathology (beta = − 0.07, *p* = 0.0034), and tau tangles (beta = − 0.30, *p* = 0.0007). TL length was not significant for any measure of AD.

For the non-AD neurodegenerative disease pathologies, mtDNAcn was positively associated with hippocampal sclerosis and the cortical clock positively associated with Lewy bodies. Among the cerebrovascular pathologies, mtDNAcn was positively associated with macroscopic infarctions and CAA. No associations with CVD pathologies were found with the cortical clock and TL was not associated with any of the non-AD neurodegenerative or CVD pathologies.

### Multimodal aging model shows that the combination of acceleration in both mtDNAcn and epigenetic age are associated with clinical outcomes and pathological indices

**Figure 2:**
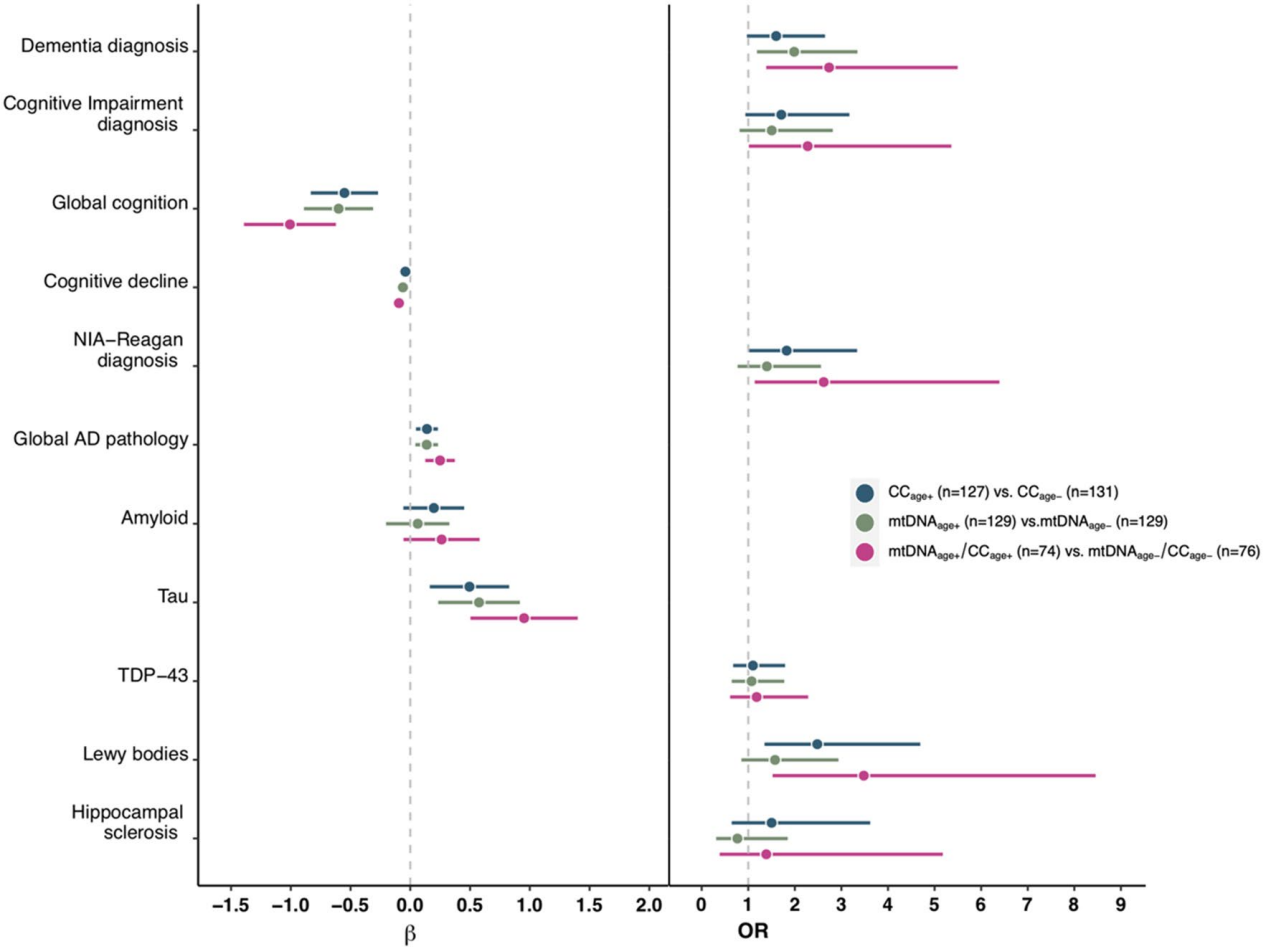
Association between binary age acceleration based on mtDNAcn and cortical clock age and clinical outcomes and pathologies. To show the impact of age acceleration on multiple aging measures, three comparisons are shown: individuals with age acceleration on mtDNA vs. those without acceleration on mtDNA in green, individuals with age acceleration on cortical clock vs. those without acceleration on cortical clock in blue and individuals with age acceleration on both mtDNA and cortical clock vs. those with age acceleration on neither in purple. Forest plots show effect sizes and confidence intervals presented as β coefficients for quantitative traits (**left panel**) and Odds Ratios for categorical (**right panel**).

To build upon the above observation that mtDNAcn and Cortical clock have independent effects on clinical outcomes and pathological indices when modeled together, we looked at multi-modal brain aging (i.e. age acceleration on multiple measures). A binary age predictor that combined CC_age_ and mtDNA_age_ had a stronger relation with most traits compared to either CC_age_ or mtDNA_age_ alone (Fig. 2). For example, individuals with age acceleration on both measures CC_age+_/mtDNAC_age+_ together have a stronger association with global cognition (beta = − 1.01, *p* = 7.8 × 10^–7^) than either CC_age+_ (beta = − 0.55, *p* = 1.5 × 10^–4^) or mtDNAC_age+_ (beta = − 0.60, *p* = 6.0 × 10^–5^). The multi-modal aging models did not show departure from additivity for any outcomes except tau tangles. The effect of CC_age+_/mtDNA_age+_ age acceleration on tau tangles was greater than twice the effect of age acceleration on only one parameter, indicated by a statistically significant likelihood ratio test (*p* = 0.018) (Supplemental Table 1). As with the multivariate model above where in the inclusion of all three measures of biological brain aging TL was no longer significant, in the multi-modal definition we found that including TL age acceleration (TL_age+/−_) did not add additional information beyond CC_age+_/mtDNAC_age+_ (data not shown).

## Discussion

We bioinformatically estimated TL from DLPFC brain samples. Most previous studies assessing the role of TL in AD have been in small sample size populations using leukocyte cells^27–31^. We assessed three biologic markers of aging independently and in a multi-modal aging model. We are not aware of other studies bringing together multiple genomic markers of biologic age measured directly in brain tissue for clinical or neuropathologic phenotypes.

We show, in DLPFC postmortem brain tissue, that longer TL has a statistically significant relationship with lower levels of β-amyloid, less hippocampal sclerosis, and more atherosclerosis. However, once other measures of brain aging are accounted for, these TL-specific relations are no longer observed. In the joint analysis including all three estimates of biologic age, higher mtDNAcn was associated with lower odds of dementia and MCI, higher global level of and change in cognition, tau tangles but only modestly associated with other pathologies. Tauopathies are known to occur without β-amyloid^60^. Cortical clock age was associated with all four AD pathologies but only had a modest association with global cognition, the continuous measure with the greatest power. We suspect that might be due to having more power for intermediate pathologic AD phenotypes compared to their downstream consequences on cognition as we previously reported^61^. Grouping multiple genomic measures of aging to create a binary variable predicting accelerated age has the largest association with clinical dementia-related outcomes and neuropathologic traits.

The *APOE* genotype has a known relationship with neuropathic outcomes. In previous ROSMAP studies, the *APOE* e4 allele was shown to be associated with lower mtDNAcn except when AD pathology was included as a covariate^34^. We also found previously a 50% greater likelihood of *APOE* e4 genotype with greater cortical clock age (OR = 1.49)^36^. Here, we did not find differences in TL by *APOE* e3 carrier status; carriers of the *APOE* e3 allele did not have different TL than *APOE* e4 allele carriers (*p* value = 0.603) or *APOE* e2 allele carriers (*p* value = 0.358).

We recently showed that incidence of AD peaks in the 10^th^ decade of life with a slight decrease afterward^62^; chronological age is a major risk factor of AD. We have also shown that genomic measures of aging are a risk factor, we reported associations between mtDNAcn and higher Cortical clock age and common neuropathologies^34,36^. The present results are consistent with this prior work, but we expand the findings by showing that brain age acceleration on multiple measures of aging, mtDNAcn and cortical clock age, has a stronger relation with outcomes than either measure individually.

While each measures brain aging, and while they are often correlated, mtDNAcn, epigenetic age and telomere length may each capture different underlying mechanisms of aging. The inverse correlation between age and mtDNAcn that has been documented in blood^63^ may be a feature of cell composition and attenuated after accounting for the contribution of platelets^64^. The regulation of mtDNAcn within a cell occurs to meet metabolic demands of the cell resulting in a range of mtDNAcn depending on tissue type and pathological conditions^65^. Lower mtDNAcn could be driven by certain pathologies rather than aging^34^. In contrast, the cortical age clock has a clear correlation with chronologic age, as it was designed using chronologic age as the benchmark for selecting CpG sites into the clock signature^35,36^. Distinct epigenetic changes that occur with aging, including DNA hypomethylation with CpG island hypermethylation, influence subsequent aging and longevity^66^. However, epigenetic modifications have not been established as causal in the process of brain aging^36^. While brain TL is itself associated with some outcomes, this measure of brain aging does not add independent information above that captured by mtDNAcn and cortical age. Gene co-expression network analysis of AD could be used to explore genomic mechanisms of aging. In blood, genes associated with two DNAm clocks and two measures of TL were associated with gene pathways that may suggest the underlying mechanism of biological aging in AD^67^.

This study does have limitations in sample size, and in that TL is bioinformatically captured which may impact the power to study the brain TL associations. We did not find TL to be associated with chronologic age or gender. Previous studies have linked leukocyte telomere length to age; however, brain TL is highly dependent on tissue type and cell composition^68–70^. Previous studies have largely reported longer TL in females. However, the length difference is not consistent and is varied across TL measurement assays^71,72^. Extensions to laboratory assays or polygenic risk scores for TL can be considered to address this issue. A parallel approach to polygenic risk scores which assigns differential weight to each biologic marker of aging could also be assessed as a future analysis. Additionally, since the analyses are cross-sectional, it does not differentiate whether the biologic processes of the genomic markers of aging are predictors or consequences of clinical or pathologic traits. We also did not have history of medication usage in study participants which may influence the relationship between the biologic predictors of age and trait outcomes. Nonetheless, this does offer some early insight into the importance of considering brain aging on multiple biological age predictors, and suggests that while these can be themselves correlated, they have independent aging related effects and potentially different biology as it relates to AD/ADRD pathogenesis.

### Supplementary Information

Supplementary Figure 1.

Supplementary Table 2.

## Data Availability

The datasets used and/or analyzed during the current study available from the corresponding author on reasonable request. Further inquiries may be directed to the corresponding author. ROSMAP resources can be requested at https://www.radc.rush.edu.
