# Supplementary material for: Evaluating genomic signatures of aging in brain tissue as it relates to Alzheimer’s disease
Source: Sci Rep. 2023 Sep 7;13:14747. doi: 10.1038/s41598-023-41400-1 (PMC10484923; doi:10.1038/s41598-023-41400-1)

Supplemental Figure 1. Joint analysis evaluation the association of TL, mtDNAcn, and cortical clock age with clinical and pathologic phenotypes adjusting for neuronal proportions from methylation data in N=258 DLPFC brain samples.. Forest plots show effect sizes and confidence intervals presented as β coefficients for quantitative traits (**left**) and Odds Ratios for categorical (**right**).

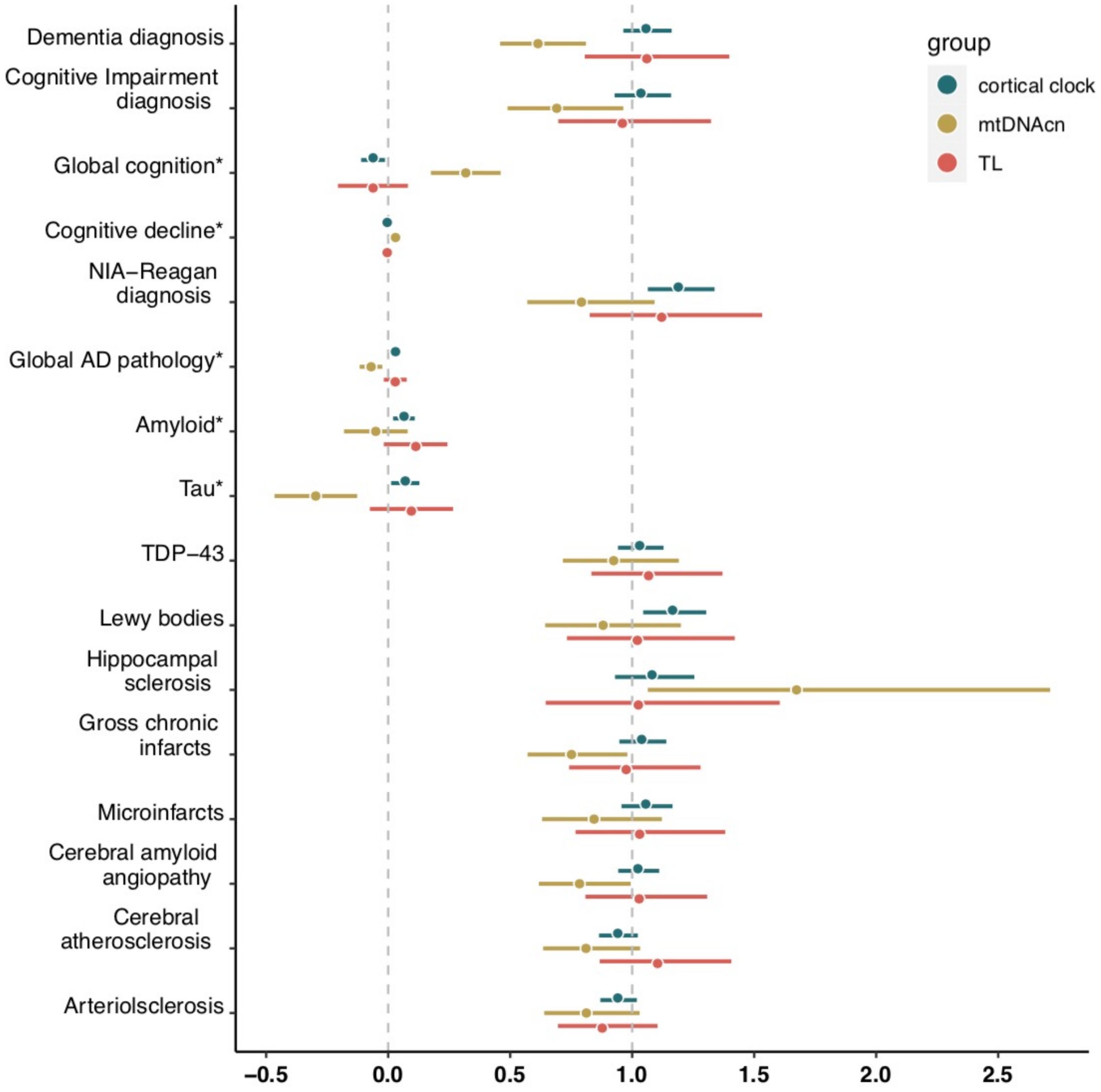

Supplement: Supplementary file 1 — Supplementary Figure 1. [file 41598_2023_41400_MOESM1_ESM.pdf]
